# Co-expression of Lgr5 and CXCR4 characterizes cancer stem-like cells of colorectal cancer

**DOI:** 10.18632/oncotarget.13214

**Published:** 2016-11-08

**Authors:** Weidong Wu, Jun Cao, Zhengyi Ji, Jingjue Wang, Tao Jiang, Honghua Ding

**Affiliations:** ^1^ Department of Gastrointestinal Surgery, Shanghai General Hospital, Shanghai Jiao Tong University School of Medicine, Shanghai 200080, China; ^2^ Department of Oncology, Shanghai General Hospital, Shanghai Jiao Tong University School of Medicine, Shanghai 200080, China

**Keywords:** cancer stem cells (CSCs), colorectal cancer (CRC), Lgr5, CXCR4

## Abstract

Therapies designed to target cancer stem cells (CSCs) in colorectal cancer (CRC) may improve treatment outcomes. Different markers have been used to identify CSCs or CSC-like cells in CRC, but the enrichment of CSCs using these markers has yet to be optimized. We recently reported the importance of Lgr5-positive CRC cells in cancer growth. Here, we studied the possibility of using Lgr5 and CXCR4 as CSC markers for CRC. We detected high Lgr5 and CXCR4 levels in stage IV CRC specimens. Both high Lgr5 and CXCR4 levels were associated with poor prognosis in stage IV CRC patients. *In vitro*, Lgr5+CXCR4-, CXCR4+Lgr5- and Lgr5+CXCR4+ cells were purified in human CRC cell lines and examined for their CSC properties. We found that compared to the unsorted cells, CXCR4+Lgr5-, Lgr5+CXCR4-, and Lgr5+/CXCR4+ cells showed significantly greater cancer mass after subcutaneous transplantation, greater tumor sphere formation, higher resistance to chemotherapy, and higher incidence of tumor formation after serial adoptive transplantation into NOD/SCID mice. Taken together, our data suggest that the combined use of Lgr5 and CXCR4 may facilitate the enrichment of CSCs in CRC, and that treating Lgr5+/CXCR4+ CRC cells may improve the outcome of CRC therapy.

## INTRODUCTION

Colorectal cancer (CRC) is one of the leading causes of cancer-related deaths worldwide [1–3], but the mechanisms regulating tumorigenesis have yet to be elucidated. Of note, the recent discovery of cancer stem cells (CSCs) has important implications for the development of novel therapies for CRC [4].

CSCs have characteristics of stem cells, are tumorigenic, and are responsible for cancer relapse and metastasis [5–8]. Treatments targeting CSCs are believed to improve current therapies for rapidly growing and highly metastatic cancers [5–8]. Although cell surface markers are generally used for isolation of CSCs by flow cytometry, none of these CSC-markers has been found to be 100% specific. These CSC-markers actually just enrich CSCs from a certain tumor, rather than purify CSCs. Hence, most characterized “CSCs” are actually CSC-like cells [9–13]. The gold standard for identifying CSCs or CSC-like cells is tumor sphere formation and tumor formation in serial adoptive transplantation.

Among all CSC surface markers, CXCR4, which is a unique receptor for stromal cell -derived factor-1 (SDF-1), has been shown to be particularly important, since the CXCR4/SDF-1 axis mediates the chemo-attractive effects that allow cancer cells to detach, migrate and seed to distal tissue [14–18]. Thus, CXCR4 has been used to characterize CSCs in renal, gastric, glioma, hepatic and breast cancers. However, CXCR4 alone appears insufficient to purify real CSCs, and is therefore used together with other markers to characterize CSCs [14, 15]. Interestingly, Zhang et al. recently showed that CXCR4 could be used as a CSC marker together with CD133 to characterize CSCs in CRC [19].

The Wnt target gene Lgr5 is a stem cell marker of the intestinal epithelium [20, 21] and the hair follicle [22, 23]. In the stem cell niche of the intestinal crypt and hair follicle, Lgr5 is specifically expressed in actively cycling cells. Transplantation and lineage tracing experiments have demonstrated that these Lgr5-positive cells maintain all cell lineages of the intestine and the hair follicle over long periods of time, and can build new intestinal tissue and hair follicles [20, 21, 24], respectively. Moreover, Lgr5-positive follicle stem cells have been shown to contribute to the formation of papillomavirus-induced tumor in the epidermis [25, 26]. Furthermore, Lgr5 has been shown to be expressed in CRC cells and has been used as a CSC marker [27–31]. However, using Lgr5 alone as a CRC CSC marker is not sufficient for isolating highly purified CSCs in CRC tissue. Additional markers are needed to further enrich the purification of CSCs or CSC-like cells from CRC.

In the current study, we addressed these questions as a follow-up study of our recent report, which demonstrates the importance of Lgr5-positive CRC cells in cancer growth [27]. Moreover, we measured the efficacy of using Lgr5, CXCR4, or both as CSC markers for CRC.

## RESULTS

### High Lgr5 and CXCR4 levels in CRC specimens are associated with poor prognosis

**Figure 1:**
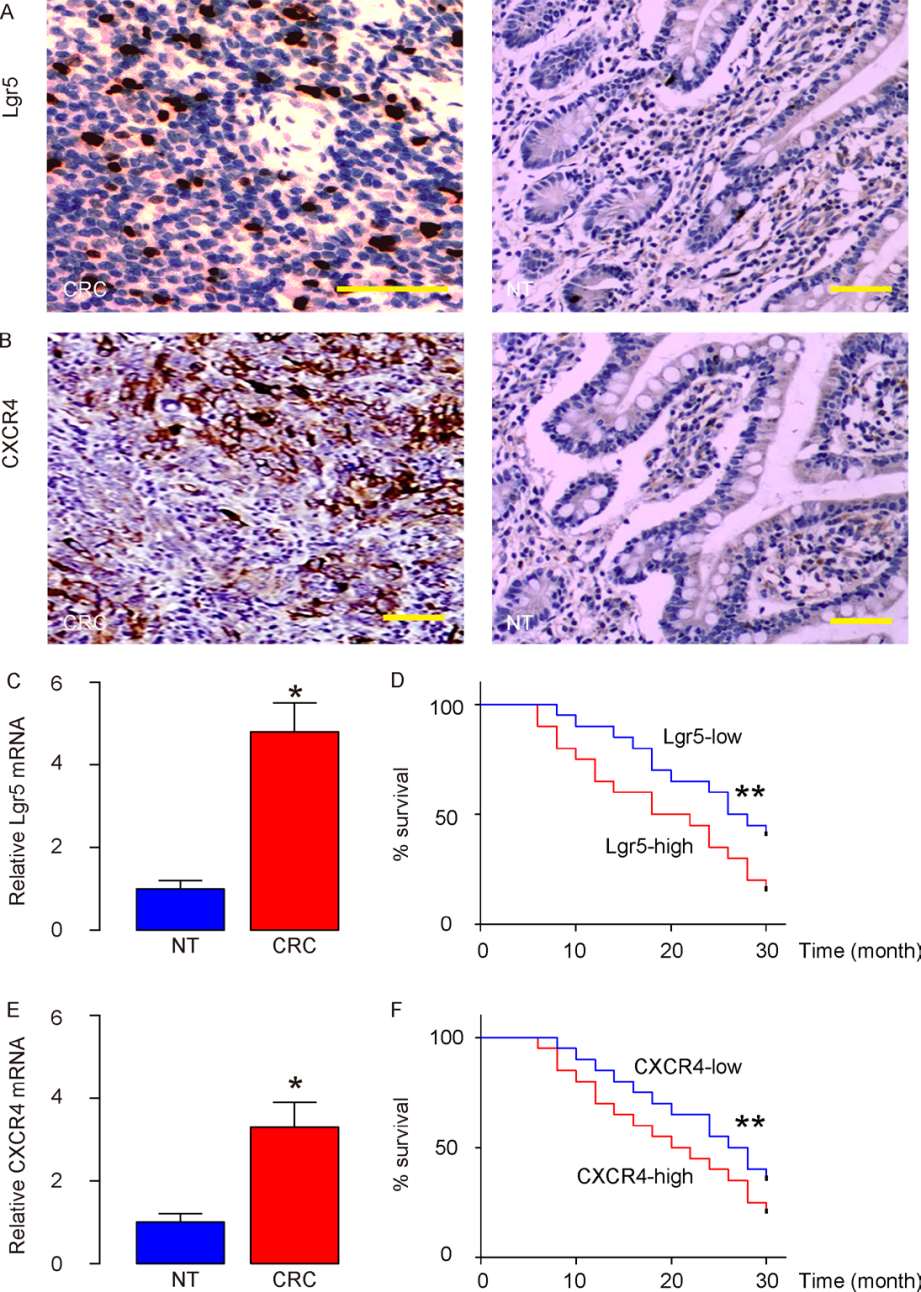
High Lgr5 and CXCR4 levels in CRC specimens are associated with poor prognosis We examined Lgr5 and CXCR4 mRNA in 80 resected Stage IV CRC specimens, and compared to the paired adjacent normal tissue (NT). (**A**–**B**) Representative IHC images for Lgr5 in CRC and NT tissue (A) and for CXCR4 in CRC and NT tissue (B) in the CRCs. (**C**) The mRNA levels of Lgr5 in the CRC tissue, compared to NT. (**D**) The 80 CRC patients were followed-up for 60 months after resection of the primary cancer. The median value of all 80 cases was chosen as the cutoff point for separating Lgr5-high cases (*n* = 40) from Lgr5-low cases (*n* = 40). Kaplan-Meier curves were analyzed for Lgr5 levels. (**E**) mRNA levels of CXCR4 in the CRC tissue, compared to NT. (**F**) The 80 CRC patients were followed-up for 60 months after resection of the primary cancer. The median value of all 80 cases was chosen as the cutoff point for separating CXCR4-high cases (*n* = 40) from CXCR4-low cases (*n* = 40). Kaplan-Meier curves were analyzed for CXCR4 levels. *p < 0.05. **p < 0.01. N = 80. Scale bars are 50 μm.

We examined Lgr5 and CXCR4 mRNA levels in 80 resected CRC (stage IV) specimens, compared to the paired adjacent normal tissue (NT) (Table 1). All patients underwent routine surgery to remove the original tumor, and no metastatic lesion was resected. We found that CRC tissue expressed high levels of Lgr5 (Figure 1A) and CXCR4 (Figure 1B), by immunohistochemistry. By RT-qPCR, we detected significantly higher levels of Lgr5 mRNA in CRC, compared to NT (Figure 1C). To examine the clinical significance of Lgr5 levels or CXCR4 levels in CRC, the 80 CRC patients were followed-up for 60 months after resection of the primary cancer. Overall survival, which was defined as the time from randomization to death, and preset as 5 years, was evaluated. The relationship of Lgr5 expression and clinicopathological characteristics was evaluated using multivariate Cox regression analysis, which showed that both were significantly associated with survival in CRC patients (Table 2). Kaplan-Meier curves were then assembled, and showed that CRC patients with high Lgr5 levels or high CXCR4 levels had a significantly worse 5-year survival than those with low Lgr5 levels or low CXCR4 levels (Figure 1D–1F). Together, these data suggest that high Lgr5 and CXCR4 levels in CRC specimens may correlate with poor prognosis.

**Table 1:** Clinicopathologic parameters of the patients (total)

	Patients (*n*; %)	*P* values
CRC tissue/Normal tumor-adjacent tissue	80 (100%)/80 (100%)	
Age (< 60/≥ 60 years old)	48 (60%)/32 (40%)	0.75
Gender (male/female)	56 (70%)/24 (30%)	0.68
Tumor site (colon)	80 (100%)	
Tumor grade (well or moderate/poor)	32 (40%)/24 (30%)/24 (30%)	0.005
Tumor stage (I/II/III/IV)	0 (0%)/0 (0%)/0 (0%)/80 (100%)	0.007
Lymph node metastasis (no/yes)	12 (15%)/68 (85%)	0.04
Distant metastasis (no/yes)	68 (85%)/12 (15%)	0.01

**Table 2:** Analysis of the prognostic values of Lgr5 or CXCR4 in CRC patients by Cox regression model

	HR	95% Cl	*P* value
Lgr5 (high vs low)	5.46	2.56–11.67	0.002
CXCR4 (high vs low)	5.38	2.42–9.13	0.002

### Preparation of Lgr5+/CXCR4-, CXCR4+/Lgr5- and Lgr5+/CXCR4+ CRC cells

**Figure 2:**
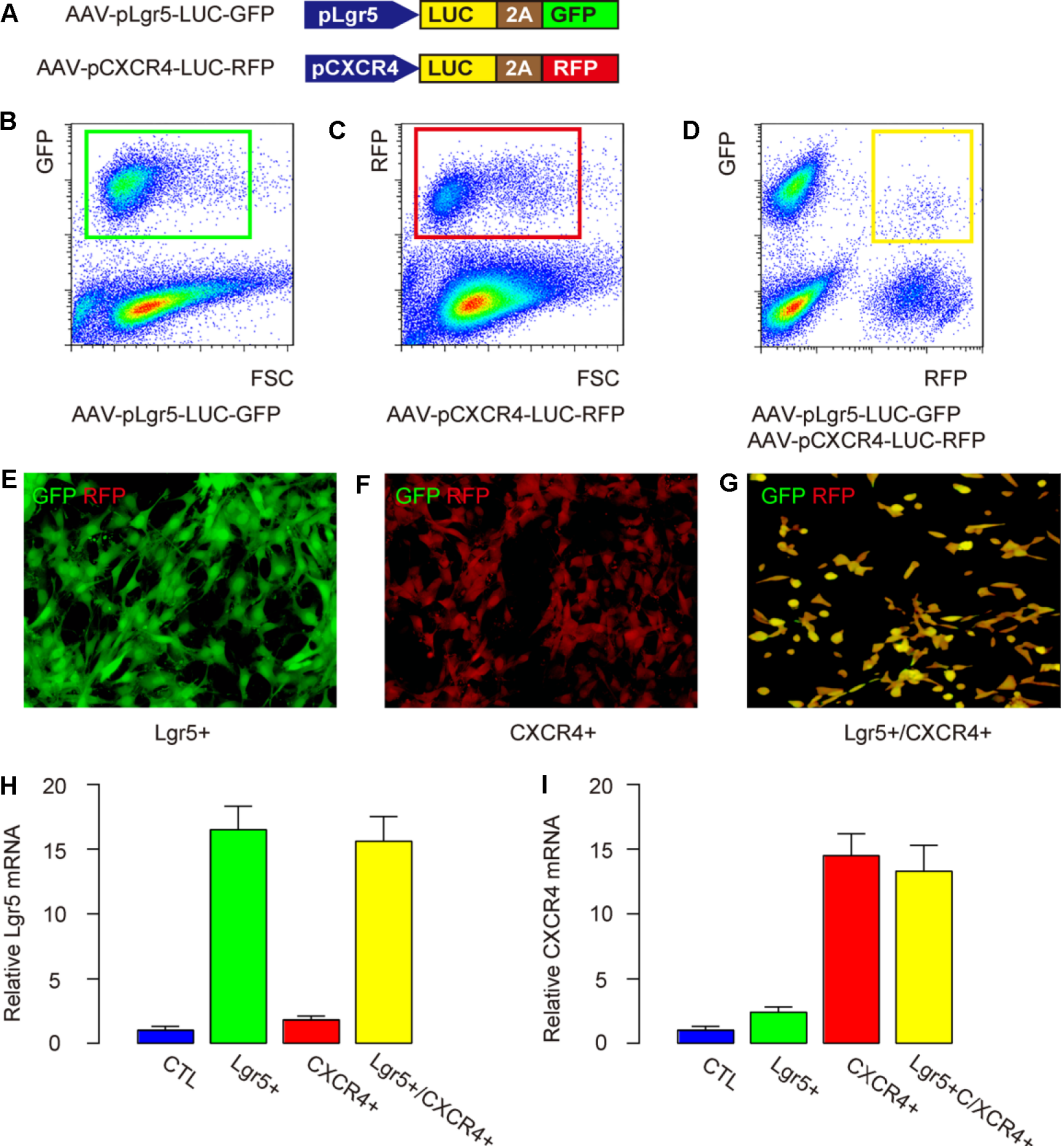
Preparation of Lgr5+/CXCR4-, CXCR4+/Lgr5- and Lgr5+/CXCR4+ CRC cells (**A**) Illustration of two AAVs (AAV-pLgr5-LUC-GFP and AAV-pCXCR4-LUC-RFP) for transduction of a CRC cell line, Caco-2. (**B**) The Lgr5+ cancer cells were isolated after transduction with AAV-pLgr5-LUC-GFP expressing both luciferase (LUC) and a GFP reporter, shown by a representative flow chart. (**C**) The CXCR4+ cancer cells were isolated after transduction with AAV-pCXCR4-LUC-RFP expressing both LUC and an RFP reporter, shown by a representative flow chart. (**D**–**G**) The Lgr5+/CXCR4+ cancer cells were co-transduced with two AAVs, shown by a representative flow chart (D). (E) The isolated Lgr5+/CXCR4- CRC cells appeared green in culture. (F) The isolated CXCR4+/Lgr5- CRC cells appeared red in culture. (G) The isolated Lgr5+/CXCR4+ CRC cells appeared yellow (both green and red) in culture. (H-I) The mRNA levels of Lgr5 (**H**) and CXCR4 (**I**) 4 in transduced cells.

In order to examine the potential of using Lgr5, CXCR4 or both as CSC markers for CRC, and to trace the cells *in vivo* in mice, we prepared two AAVs for transduction of a CRC cell line, Caco-2. The first AAV is AAV-pLgr5-LUC-GFP, and the second AAV is AAV-pCXCR4-LUC-RFP (Figure 2A). The Lgr5+ cancer cells transduced with AAV-pLgr5-LUC-GFP expressed both luciferase (LUC) and GFP reporter. The transduced Lgr5+ cells (transduction efficiency of 83.7 ± 5.9%) were purified by flow cytometry based on GFP expression, and were traced *in vivo* by LUC (Figure 2B). The CXCR4+ cancer cells transduced with AAV-pCXCR4-LUC-RFP expressed both luciferase (LUC) and an RFP reporter. The transduced CXCR4+ cells (transduction efficiency of 85.5 ± 6.5%) were purified by flow cytometry based on RFP expression, and were traced *in vivo* by LUC (Figure 2C). The Lgr5+/CXCR4+ cancer cells were generated by co-transduction with both AAVs. The transduced Lgr5+/CXCR4- cells, CXCR4+/Lgr5- cells, Lgr5+/CXCR4+ cells (transduction efficiency for double viruses was 72.2 ± 6.1%) were purified by flow cytometry based on RFP and GFP co-expression, and were traced *in vivo* by LUC (Figure 2D). The purified Lgr5+/CXCR4- CRC cells appeared green in culture (Figure 2E). The purified CXCR4+/Lgr5- CRC cells appeared red in culture (Figure 2F). The purified Lgr5+/CXCR4+ CRC cells appeared yellow (both green and red) in culture (Figure 2G). Moreover, the mRNA levels of Lgr5 (Figure 2H) and CXCR4 (Figure 2I) confirmed the enrichment of Lgr5 and/or CXCR4 in these cells.

### Lgr5+/CXCR4+ cells generate the greatest cancer mass after s.c. transplantation

**Figure 3:**
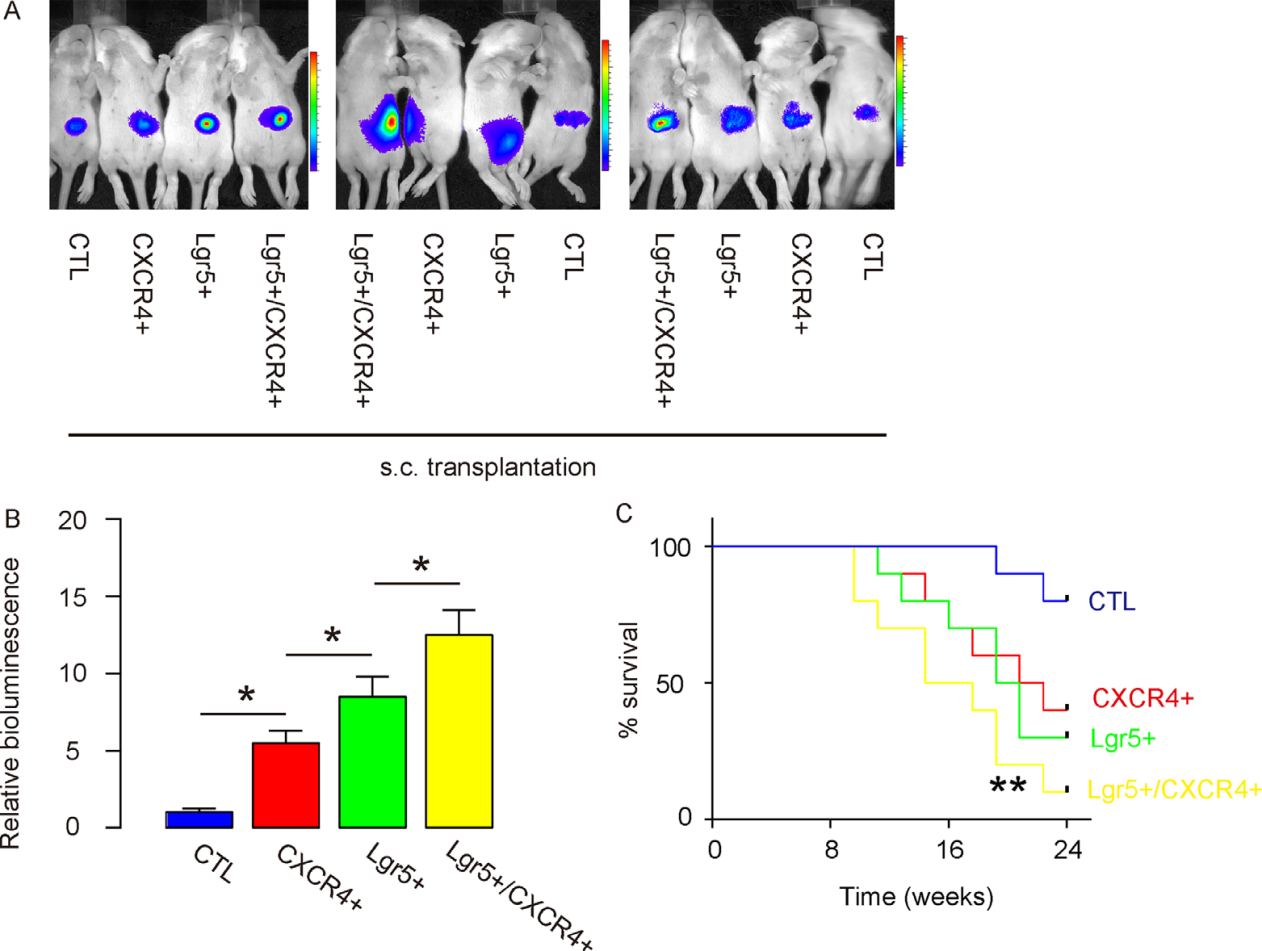
Lgr5+/CXCR4+ cells generate the greatest cancer mass after s.c transplantation. The same number of control (unpurified, transduced with LUC), CXCR4+/Lgr5-, Lgr5+/CXCR4- and Lgr5+/CXCR4+ cells were s.c. implanted into NOD/SCID mice. (**A**–**B**) The mass of the generated tumor was analyzed based on bioluminescence examination, shown by quantification (B), and by representative images (A). (**C**) The survival curve of the mice that had received transplantation of unsorted control cells, CXCR4+/Lgr5-, Lgr5+/CXCR4- and Lgr5+/CXCR4+ cells for 24 weeks. *p < 0.05. *n* = 10.

Thus, the same number of control (unpurified, transduced with LUC), CXCR4+/Lgr5-, Lgr5+/CXCR4- and Lgr5+/CXCR4+ Caco-2 cells were s.c. implanted into NOD/SCID mice. We found that, compared to unsorted control cells, CXCR4+/Lgr5-, Lgr5+/CXCR4- and Lgr5+/CXCR4+ cells generated tumors with significantly increased mass 8 weeks after transplantation; likewise, the Lgr5+/CXCR4+ cells generated the greatest tumor mass among all, based on bioluminescence examination, shown by representative images (Figure 3A), and by quantification (Figure 3B). Next, we evaluated the survival of the mice that had received transplantation of unsorted control cells, CXCR4+/Lgr5-, Lgr5+/CXCR4- and Lgr5+/CXCR4+ cells. We found that the mice that received Lgr5+/CXCR4+ cells had the shortest survival (Figure 3C).

### Lgr5+/CXCR4+ cells generate more tumor spheres *in vitro*

**Figure 4:**
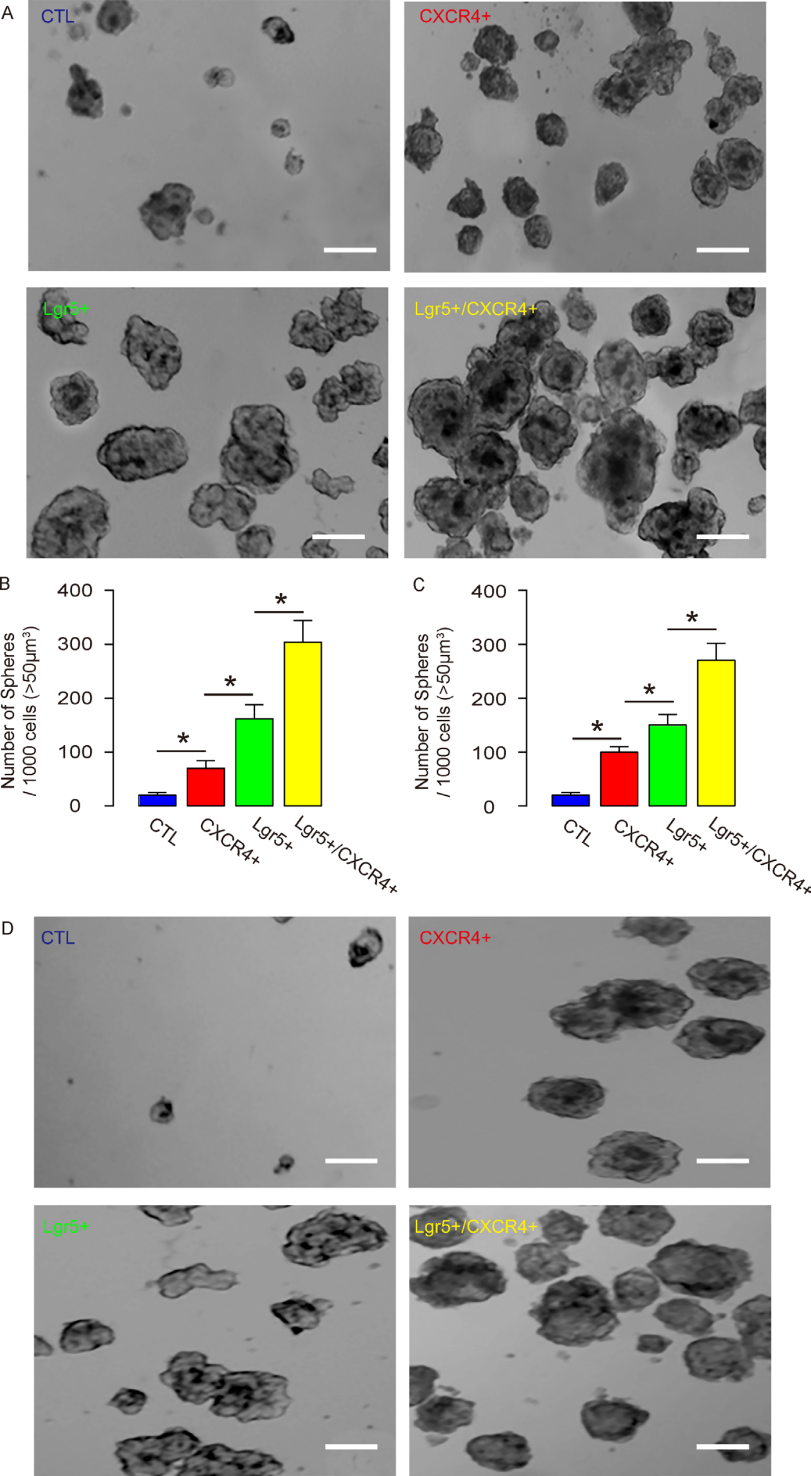
Lgr5+/CXCR4+ cells generate more tumor spheres *in vitro* The control, CXCR4+/Lgr5-, Lgr5+/CXCR4- and Lgr5+/CXCR4+ Caco-2 and HT-29 cells were subjected to tumor sphere formation assay. (**A**–**B**) The formation of tumor sphere in Caco-2 cells was analyzed, shown by representative images (A), and by quantification (B). (**C**–**D**) The formation of tumor sphere in HT-29 cells was analyzed, shown by quantification (C) and by representative images (D). *p < 0.05. *n* = 10. Scale bars are 50 μm.

Two human CRC lines were used. The control, CXCR4+/Lgr5-, Lgr5+/CXCR4- and Lgr5+/CXCR4+ Caco-2 and HT-29 cells were subjected to tumor sphere formation assay. We found that, compared to unsorted control cells, CXCR4+/Lgr5-, Lgr5+/CXCR4- and Lgr5+/CXCR4+ Caco-2 and HT-29 cells generated significantly more tumor spheres. Moreover, the Lgr5+/CXCR4+ cells generated significantly more tumor spheres than CXCR4+/Lgr5- and Lgr5+/CXCR4- cells (Figure 4A–4D). Hence, Lgr5+/CXCR4+ cells are a more enriched CSC population than Lgr5+ or CXCR4+ cells alone.

### Lgr5+/CXCR4+ cells are more resistant to chemotherapy

**Figure 5:**
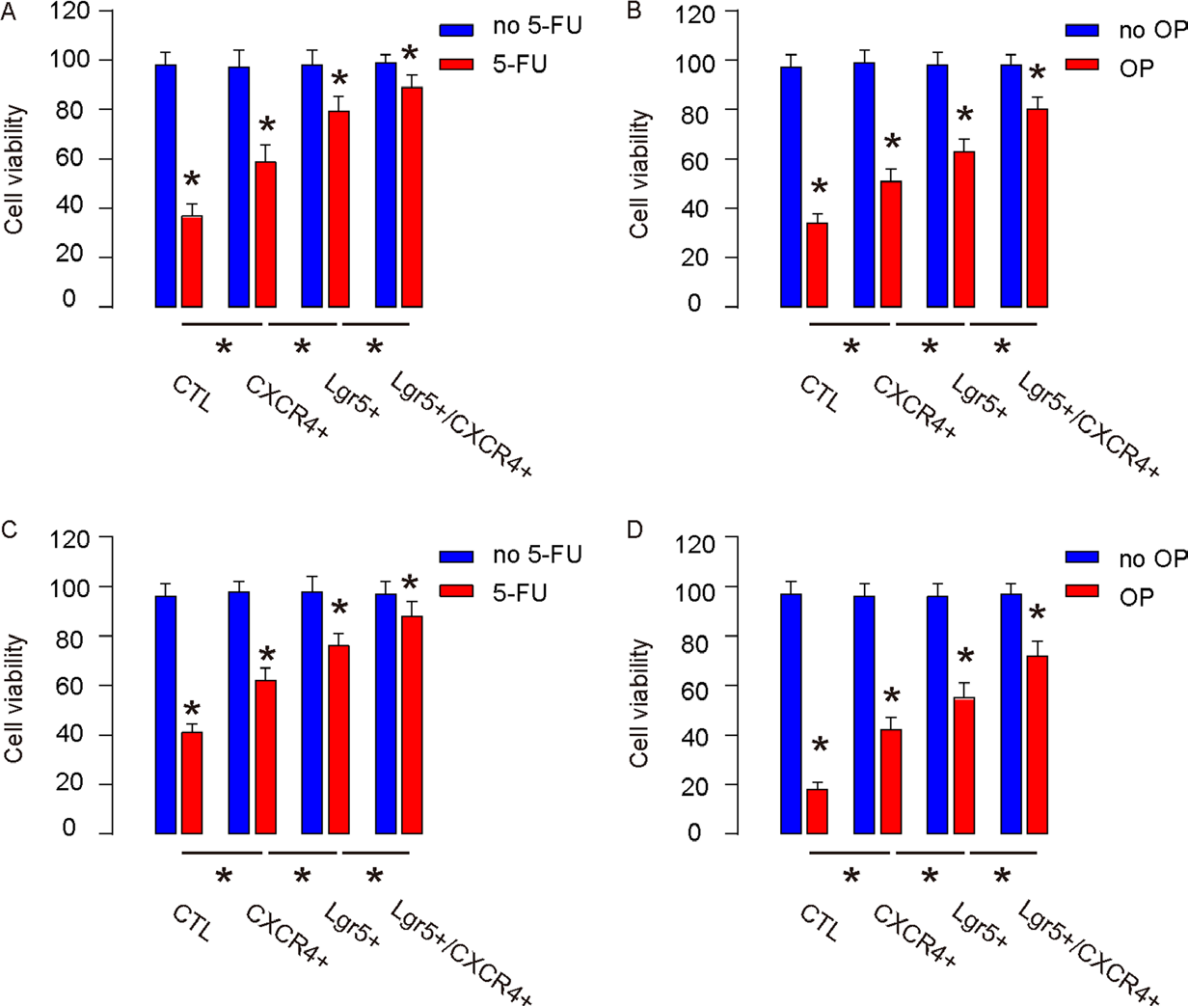
Lgr5+/CXCR4+ cells are more resistant to chemotherapy The control, CXCR4+/Lgr5-, Lgr5+/CXCR4- and Lgr5+/CXCR4+ Caco-2 and HT-29 cells were subjected to 5 μmol/l 5-FU or 15 μmol/l Oxaliplatin (OP). (**A**–**B**) The cell viability of Caco-2 by 5-FU (A) and by OP (B) was analyzed 48 hours later in a CCK-8 assay. (**C**–**D**) The cell viability of HT-29 by 5-FU (C) and by OP (D) was analyzed 48 hours later in a CCK-8 assay. *p < 0.05. *n* = 10.

Next, the control, CXCR4+/Lgr5-, Lgr5+/CXCR4- and Lgr5+/CXCR4+ Caco-2 and HT-29 cells were subjected to 5-FU or Oxaliplatin (OP) treatment *in vitro*. We found that, compared to unsorted control cells, CXCR4+/Lgr5-, Lgr5+/CXCR4- and Lgr5+/CXCR4+ cells appeared to have higher cell viability, and that Lgr5+/CXCR4+ cells were most resistant to either treatment in a CCK-8 assay (Figure 5A–5D). Hence, Lgr5+/CXCR4+ cells are more resistant to chemotherapy.

### Lgr5+/CXCR4+ cells induce the highest occurrence of tumor formation in serial adoptive transplantation

**Figure 6:**
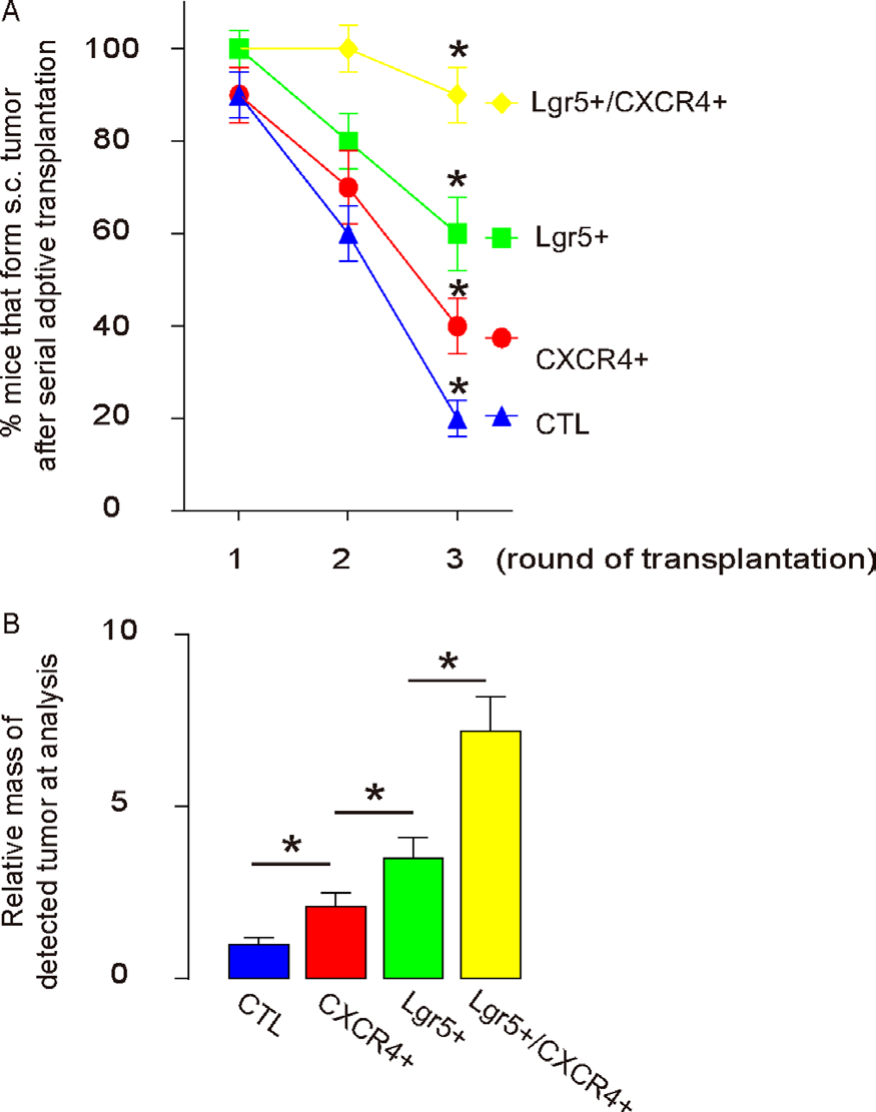
Lgr5+/CXCR4+ cells induce the highest occurrence of tumor formation in serial adoptive transplantation For serial transplantation, 20 tumor cells were isolated from s.c. tumor developed from either control, CXCR4+/Lgr5-, Lgr5+/CXCR4- and Lgr5+/CXCR4+ cells transplanted s.c. into new NOD/SCID mice. Tumor formation was examined by bioluminescence after 6 weeks and then confirmed by histology of the dissected tissue. The newly formed tumors were then dissected out and used for isolation of 20 tumor cells for the next round of transplantation. Three rounds of transplantation were performed. (**A**–**B**) The rate of tumor formation (A) and the average formed tumor size (B) were quantified. *p < 0.05. *n* = 10.

Another gold standard for determining CSC-like cells is their potential for tumor formation after serial adoptive transplantation. Thus, 20 tumor cells isolated from either control, CXCR4+/Lgr5-, Lgr5+/CXCR4- or Lgr5+/CXCR4+ cells were transplanted s.c. into new NOD/SCID mice. Tumor formation was verified by bioluminescence after 6 weeks, and then confirmed by histology of the dissected tissue. From this tissue, we then isolated 20 tumor cells for the next round of transplantation. Three rounds of transplantation were performed. We found that Lgr5+/CXCR4+ cells had significantly higher rates of tumor development, compared to others, based on bioluminescence examination (Figure 6A). Moreover, the tumor mass formed by Lgr5+/CXCR4+ cells was significantly greater than tumor mass from the other cell populations (Figure 6B). These data suggest that Lgr5+/CXCR4+ cells are highly enriched for CSCs in CRC, and are better markers for sorting CSC-like cells, compared to Lgr5+ cells alone.

## DISCUSSION

The importance of CSCs to CRC growth and metastasis has been well documented. In the past, identification of CSCs has largely relied on flow-cytometry-based examination of CD133, side population and high aldehyde dehydrogenase (ALDH) activity. However, increasing evidence has called these methods into question. For example, CD133+ cells are not all CSCs [32]. Also, ALDH activity is detected in non-stem/progenitor and non-cancer cells [33, 34]. Hence, isolation of CSCs with these methods is not ideal. In CRC, Lgr5 has been shown to be expressed in CRC cells and can be used as a CSC marker [27–31]. In our previous work, we showed that elimination of Lgr5+ cells in CRC nearly completely inhibited growth of CRC both *in vitro* and *in vivo*. Thus, Lgr5 may be a marker that is expressed in all CSCs in CRC. However, using Lgr5 alone as a CRC CSC marker is not sufficient to isolate highly purified CSCs in CRC tissue.

The SDF-1/CXCR4 axis is a potential chemoattractant system that regulates cell migration and homing, and plays an important and unique role in the regulation of stem/progenitor/cancer cell trafficking. CXCR4 is known to be expressed on some tumor cells, which may metastasize to the organs that secrete/express SDF-1 [35–39]. SDF-1 exerts pleiotropic effects regulating metastasis-associated processes, including cancer cell locomotion, chemoattraction and adhesion, and tumor vascularization, which are all related to CSC properties [35–39]. Importantly, the purification of CSCs using the CXCR4 marker alone is less efficient [40–46]. Recently, Zhang et al. showed that CXCR4 could be used as a CSC marker together with CD133 to characterize CSCs in CRC [19]. These previous studies highlight the importance of using a combination of cell markers to identify CSCs. Nevertheless, the extent to which a combination of Lgr5 and CXCR4 may improve the enrichment of CSCs in CRC has not been examined.

In the current study, we purified CXCR4 and Lgr5 double positive cells, which comprise a sub-population of Lgr5+ cells among all CRC cells. We found that these Lgr5+/CXCR4+ cells showed higher tumor formation potential *in vitro* and *in vivo*, were more resistant to chemotherapy, and had a greater tumor-generating ability after serial adoptive transplantation. These are gold standard measurements for determining CSC properties. Here, all three assays supported Lgr5+/CXCR4+ cells as a highly purified CSC population, and demonstrated these cells' advantage over Lgr5+ cells alone in identifying real CSC populations in CRC. Accurate identification, in turn, promotes the development of innovative, targeted therapy.

In a previous study, CXCR4+ cells were shown to express high levels of Lgr5, which is a known marker of gastric epithelial stem cells located in normal mucosal glands [47]. Lgr5 controls stemness in epithelial cells lining the gut [47], while CXCR4 is critical for cell adhesion, migration and metastasis [35–39]. Thus, combined expression of these two factors meets the requirement for a CSC.

Although here we provide rigorous data to support the usefulness of a combination of Lgr5 and CXCR4 for characterizing CSCs in CRC, additional efforts are needed to confirm its value in analyzing human CRC specimens. For example, larger samples should be analyzed to detect Lgr5 and CXCR4 expression not only in primary CRCs, but also in metastatic tumors.

## MATERIALS AND METHODS

### Protocol approval

All the experimental methods have been approved by the research committee at Shanghai General Hospital. All animal experiments were approved by the Institutional Animal Care and Use Committee at Shanghai General Hospital (Animal Welfare Assurance). All the experiments have been carried out in accordance with the guidelines from the research committee at Shanghai General Hospital. The methods regarding animals and human specimens were carried out in “accordance” with the approved guidelines. Surgeries were performed in accordance with the Principles of Laboratory Care, supervised by a qualified veterinarian.

### Patient specimens

Surgical specimens from 80 CRC (stage IV) patients and paired adjacent non-tumor tissues (NT) were obtained postoperatively in Shanghai General Hospital from 2007 to 2009 (Table 1). All patients gave signed, informed consent for the tissue to be used for scientific research. Ethical approval for the study was obtained from the Shanghai General Hospital. All diagnoses were based on pathological and/or cytological evidence. The histological features of the specimens were evaluated by senior pathologists according to the World Health Organization classification criteria. All patients had been followed-up for 60 months. Complete clinical data were electronically recorded.

### Cell line culture and treatment

Two human colon epithelial adenocarcinoma cell lines, Caco-2 and HT-29, were used in the current study. Caco-2 was originally developed by Dr. Jorgen Fogh, and HT-29 was obtained from a 44 year-old female, and has been described before [48]. Both lines were purchased from APCC (American Type Culture Collection, Manassas, VA, USA), and cultured in RPMI1640 medium (Invitrogen, Carlsbad, CA, USA) supplemented with 15% fetal bovine serum (FBS; Sigma-Aldrich, St Louis, MO, USA) in a humidified chamber with 5% CO_2_ at 37°C. Fluorouracil (5-FU, Sigma-Aldrich) was prepared in a stock of 1 mmol/l and applied to the cultured cells at 5 μmol/l. Cisplatin (Sigma-Aldrich) was prepared in a stock of 1 mmol/l and applied to the cultured cells at 20 μmol/l.

### Preparation of adeno-associated virus

Luciferase (LUC) allows *in vivo* tracing of cells. GFP is a green fluorescent protein and RFP is a red fluorescent protein. The pLgr5 in the AAV-pLgr5-LUC-GFP plasmid and the pCXCR4 in the AAV-pCXCR4-LUC-RFP plasmid were prepared from a full-length human Lgr5 or CXCR4 promoter, respectively. The 5′ and 3′ homology regions for the Lgr5 promoter were 1.9 kb (between -1954 from human Lgr5 transcript start and -48 from human Lgr5 transcript start) and the 5′ and 3′ homology regions for the CXCR4 promoter were 2.6 kb (between –2760 from human CXCR4 transcript start and -85 from human CXCR4 transcript start). The pLgr5 and pCXCR4 were amplified by PCR with EcoRI-restriction-endonuclease-forward and NheI-restriction-endonuclease-reverse primers, using human genomic DNA as a template. The pLgr5 construct was then subcloned into the 50-EcoRI and 30-NheI sites of the pAAV-CMV-LUC-2A-GFP vector (Clontech, Mountain View, CA, USA) to replace the CMV promoter to generate pAAV-pLgr5-LUC-GFP. The pCXCR4 construct was then subcloned into the 50-EcoRI and 30-NheI sites of the pAAV-CMV-LUC-2A-RFP vector (Clontech, Mountain View, CA, USA) to replace the CMV promoter to generate pAAV-pCXCR4-LUC-RFP. Sequencing was performed to confirm the correct orientation of the prepared pAAV-pLgr5-LUC-GFP and pAAV-pCXCR4-LUC-RFP, which were then used to generate AAV, with a packaging plasmid carrying the serotype 6 rep and cap genes and a helper plasmid carrying the adenovirus helper functions (Applied Viromics, LLC. Fremont, CA, USA), using Lipofectamine 2000 reagent (Invitrogen). The control cells were transduced with AAV generated from pAAV-CMV-LUC-2A-GFP vector. The small 2A peptide sequences, when cloned between genes, allow for efficient, stoichiometric production of discrete protein products within a single vector through a novel “cleavage” event within the 2A peptide sequence. The AAVs were purified using CsCl density centrifugation and then titration was determined by a quantitative densitometric dot-blot assay. For cell transduction *in vitro*, the cells were incubated with AAV at a MOI of 100 for 12 hours. Stable transduced cells were selected by flow cytometry based on GFP, RFP or both. Transduced cells were monitored *in vivo* by their expression of luciferase.

### Mouse manipulation

Ten week-old male NOD/SCID mice (SLAC Laboratory Animal Co. Ltd, Shanghai, China) were used for subcutaneous (s.c.) transplantation of tumor cells and serial adoptive transfer. Bioluminescence was monitored 4 weeks after s.c. transplantation. For s.c. transplantation of cancer cells into NOD/SCID mice, 500 cancer cells were implanted s.c. and tumor formation was examined after 8 weeks by bioluminescence. For serial adoptive transplantation of cancer cells, 20 cancer cells were isolated from implanted tumor and re-transplanted s.c. into NOD/SCID mice. Tumor formation was examined after 6 weeks by bioluminescence. Three rounds of serial adoptive transfer were performed.

### Tumor monitoring by bioluminescence

Formation of tumor at s.c. sites was monitored by luciferin assay, based on luciferase activity of tumor cells. Bioluminescence was measured with the IVIS imaging system (Xenogen Corp., Alameda, CA, USA). All of the images were taken 10 minutes after intraperitoneal injection of luciferin (Sigma-Aldrich) of 150 mg/kg body weight, as a 60-second acquisition and 10 of binning. During image acquisition, mice were sedated continuously via inhalation of 3% isoflurane. Image analysis and bioluminescent quantification were performed using Living Image software (Xenogen Corp.).

### Primary tumor sphere culture

Cancer cells were washed, acutely dissociated in oxygenated artificial cerebrospinal fluid and subjected to enzymatic dissociation to single cells. Afterward, single cancer cells were re-suspended in tumor sphere media (TSM) consisting of serum-free DMEM, human recombinant Epidermal growth factor (20 ng/ml; Sigma-Aldrich), bFGF (20 ng/ml; Sigma-Aldrich), leukemia inhibitory factor (10 ng/ml; Sigma-Aldrich) and N-acetylcysteine (60 μg/ml; Sigma-Aldrich), and then plated at a density of 2 × 10^4^cells/60 mm plate for examination of tumor sphere formation.

### Cell viability assay

A CCK-8 detection kit (Sigma-Aldrich) was used to measure cell viability according to the manufacturer's instructions. Briefly, cells were seeded in a 96-well microplate at a density of 5 × 10^4^/ml. After 24 h, the cells were treated with resveratrol. Subsequently, CCK-8 solution (20 ml/well) was added and the plate was incubated at 37°C for 2 h. The viable cells were counted by absorbance measurements with a monochromator microplate reader at a wavelength of 450 nm. The optical density value was reported as the percentage of cell viability in relation to the control group (set as 100%).

### Quantitative real-time PCR (RT-qPCR)

Total RNA was extracted from mouse tissue or cultured cells with an RNeasy kit (Qiagen, Hilden, Germany) for cDNA synthesis. RT-qPCR was performed in duplicates with QuantiTect SYBR Green PCR Kit (Qiagen). Primers: Lgr5 forward, 5′-GAGGATCTGGTGAGCCTGAGAA-3′, reverse, 5′-CATAAGTGATGCTGGAGCTGGTAA-3′; CXCR4 forward 5′-TCAGTGGCTGACCTCCTCTT-3′, reverse 5′-CTTGGCCTTTGACTGTTGGT-3′, β-actin forward, 5′-CAACTGGGACGACATGGAGAAA-3′, reverse, 5′-GATAGCAACGTACATGGCTGGG-3′. Data were collected and analyzed using the 2-ΔΔCt method for quantification of relative mRNA expression levels. Values of genes were first normalized against β-actin, and then compared to the experimental controls.

### Immunohistochemistry

The resected CRC tumor tissues and normal colon tissue controls were fixed in 4% PFA and cut into 4 μm slices. The sections were pretreated using an autoclave in citrate buffer (pH 6.0) for 18 min at 121°C, which were naturally cooled and were rinsed three times with PBS for 15 minutes. The slices were then treated with 3% hydrogen peroxide (H_2_O_2_) for 5 min and rinsed three times with PBS for 6 minutes. Next, sections were immunostained with anti-CXCR4 or anti-Lgr5 antibody (Abcam, ab75732, Beijing, China) and were treated with DAB for 15 min in the dark at room temperature. Finally, the sections were washed, counterstained, dehydrated and mounted. The evaluation of positivity was performed by checking the positive cell number per mm^2^ field, and the results were consistent with the RT-qPCR findings.

### Statistical analysis

All of the statistical analyses were performed using GraphPad Prism 6 (GraphPad Software, San Diego, CA, USA). Statistical analysis of group differences was carried out using a one-way analysis of variance (ANOVA) test followed by Turkey multiple comparison post-hoc analysis. The relationships of Lgr5/CXCR4 expression and clinicopathological characteristics with overall survivals were evaluated using multivariate Cox regression analysis. Patients' survival was determined by Kaplan-Meier analysis. All values represent the mean ± standard deviation (SD). A value of *p* < 0.05 was considered statistically significant after Bonferroni correction.
